# Protective Effects of Mangosteen Extract on H_2_O_2_-Induced Cytotoxicity in SK-N-SH Cells and Scopolamine-Induced Memory Impairment in Mice

**DOI:** 10.1371/journal.pone.0085053

**Published:** 2013-12-27

**Authors:** Jintana Sattayasai, Pongsatorn Chaonapan, Tarinee Arkaravichie, Rungtip Soi-ampornkul, Sarawut Junnu, Patcharakajee Charoensilp, Jutima Samer, Jiraporn Jantaravinid, Patarabutr Masaratana, Bhoom Suktitipat, Juthatip Manissorn, Visith Thongboonkerd, Neelobol Neungton, Primchanien Moongkarndi

**Affiliations:** 1 Department of Pharmacology, Faculty of Medicine, Khon Kaen University, Khon Kaen, Thailand; 2 Department of Biochemistry, Faculty of Medicine Siriraj Hospital, Mahidol University, Bangkok, Thailand; 3 Department of Microbiology, Faculty of Pharmacy, Mahidol University, Bangkok, Thailand; 4 Medical Proteomics Unit, Office for Research and Development, Faculty of Medicine Siriraj Hospital, Mahidol University, Bangkok, Thailand; Massachusetts General Hospital/Harvard Medical School, United States of America

## Abstract

Mangosteen extracts (ME) contain high levels of polyphenolic compounds and antioxidant activity. Protective effects of ME against β-amyloid peptide (Aβ), induced cytotoxicity have been reported. Here, we further studied the protective effects of ME against oxidative stress induced by hydrogen peroxide (H_2_O_2_) and polychlorinated biphenyls (PCBs), and demonstrated the protection against memory impairment in mice. The cytoprotective effects of ME were measured as cell viability and the reduction in ROS activity. In SK-N-SH cell cultures, 200 μg/ml ME could partially antagonize the effects of 150 or 300 µM H_2_O_2_ on cell viability, ROS level and caspase-3 activity. At 200, 400 or 800 µg/ml, ME reduced AChE activity of SK-N-SH cells to about 60% of the control. *In vivo* study, Morris water maze and passive avoidance tests were used to assess the memory of the animals. ME, especially at 100 mg/kg body weight, could improve the animal’s memory and also antagonize the effect of scopolamine on memory. The increase in ROS level and caspase-3 activity in the brain of scopolamine-treated mice were antagonized by the ME treatment. The study demonstrated cytoprotective effects of ME against H_2_O_2_ and PCB-52 toxicity and having AChE inhibitory effect in cell culture. ME treatment in mice could attenuate scopolamine-induced memory deficit and oxidative stress in brain.

## Introduction

Alzheimer’s disease (AD) is the most common cause of dementia affecting approximately 10-15% of elderly after 65 years of age [1]. The incidence of dementia is doubling every 5 years afterward and reaches >50% by the age over 85 years [2,3]. The situation of AD has become progressively serious as a result of a global increase in life expectancy. This fatal disease usually starts with an insidious onset of neuronal death, followed by gradually progressive impairment of brain function and finally death within approximately 10 years after the diagnosis [1,3]. At present, the definite causes of AD remain unknown, and there is no successful treatment or prevention. Two hallmarks found in AD are neurofibrillary tangles and senile plaques accumulation in the brain. β-amyloid (Aβ), the main content of senile plaques, has been proposed to associate with neuron death in AD [1,3]. Notably, the pathogenesis of AD has also been linked to acetylcholine deficiency in the brain as suggested by the death of cholinergic neurons [4,5]. Depleted acetylcholine or blockade of the central muscarinic acetylcholine receptor results in impaired learning and memory functions in both animals and humans [6,7]. Anticholinergic drugs (muscarinic blocker), such as scopolamine, have been used as potent amnesic agents. Interestingly, scopolamine-induced amnesia mouse model is commonly used for the screening of memory-enhancing and anti-amnesic drugs. Despite its known limitations, the scopolamine model of cholinergic dysfunction is recognized as a screening paradigm to assess the memory and cognition enhancing properties of substances proposed to combat age-associated decline in cognitive performance or dementia of the Alzheimer disease [8-12]. 

Furthermore, neuronal cell death in AD and dementia could be enhanced by various inflammatory processes and cellular oxidative stress through endogenous reactive oxygen species (ROS) or exogenous chemical induction [5,6]. Exogenous chemical compounds inducing oxidative stress and neurotoxicity included Polychlorinated biphenyls (PCBs) which are universal toxic environmental pollutants widely used in various industries as dielectric and coolant fluids. It has been reported that chronic exposure to PCBs can induce cellular oxidative stress and apoptosis especially in neurons resulting in progressive memory cell loss and finally dementia [11,12]. 

 Due to the central roles of acetylcholine in the pathogenesis of AD, acetylcholinesterase (AChE), the key enzyme involved in the breakdown of acetylcholine, is considered as a promising therapeutic target for AD. Acetylcholinesterase inhibitors (AChEIs) can reduce the rate at which acetylcholine (ACh) is broken down, then, increasing the concentration of ACh in the brain and combating the loss of ACh caused by the death of cholinergic neurons [4,5]. Additionally, AChEIs have also been shown to protect cells from free radical toxicity, β-amyloid-induced injury, and increase antioxidant production [4]. A potential source of AChEIs is provided by the abundance of plants in nature. Numerous phytoconstituents and promising plant species have been reported as AChEIs [13].

*Garcinia mangostana* L. (Mangosteen) is a tropical evergreen tree that grows well in Southeast Asia, particularly in Thailand. The fruit hull or pericarp of mangosteen has been used as a traditional medicine against several infections for many decades. Nearly 50 out of 200 known xanthones have been isolated in high amount from mangosteen extract (ME) [14]. Xanthones have been shown to possess strong antioxidant, anti-inflammation and antitumor activities. Interestingly, previous studies demonstrated neuroprotective effects of the water-soluble partition of ethanolic extract of mangosteen pericarp against oxidative stress in various neuronal cell models [15]. In agreement, we have previously reported protective effects of water-soluble ME against Aβ-induced cytotoxicity, oxidative stress and alteration of proteome in SK-N-SH cells [16]. Notably, the presence of AChEIs has not yet been studied in ME despite the abundance in numerous phytoconstituents in this plant [13]. 

The present study was therefore conducted to further explore *in vitro* protective effects of ME against H_2_O_2,_ and PCB-52, as a model of endogenous and exogenous oxidant from environmental pollutants, respectively. We determined AChE inhibitory effects of ME that could potentially improve memory impairment in AD. In addition*, in vivo* protective effects of ME were investigated in scopolamine-induced amnesia mouse model.

## Materials and Methods

### Cell culture and cytotoxicity induction by H_2_O_2_

SK-N-SH human neuroblastoma cells (HTB-11) (ATCC; Manassas, VA) were cultured in minimum essential medium (MEM) with supplemented nutrients and essential conditions, according to the manufacturer’s instructions (GIBCO, Invitrogen Corporation; Grand Island, NY). Hydrogen peroxide (H_2_O_2_) (Merck Schuchardt OHG, Hohenbrunn, Germany) was freshly diluted in phosphate buffer saline solution (PBS) before adding to the cells at final concentrations of 37.5-600 µM. PCB-52 (Accu Standard Inc., New Haven, CT) was freshly diluted in DMSO before adding to the cells at final concentrations of 5-30 µg/ml. The cells treated with H_2_O_2_ or PCB-52 were then incubated at 37°C for 24 h.

### Preparation and cytotoxicity screening of Mangosteen Extract (ME)

Mangosteen fruits were obtained from Chanthaburi province, Thailand. The pericarp was removed, dried, and then extracted with ethanol. The crude ethanolic extract was then partitioned with ethylacetate (EtOAc) and water as previously described [16]. The partially purified water partition contained total phenolic compounds that provided antioxidative properties, ranging between 150-200 mg/g of standard gallic acid equivalent (milligram of gallic acid per gram of the extract). The amount of α-mangostin that possessed a strong cytotoxicity was presented less than 2%. ME was then concentrated by rotary evaporator, and kept in desiccator at 4 °C. Each concentrated lot contained the moisture content less than 5%, no contamination of insecticides, toxic heavy metals or any toxic chemicals and no detectable EtOAc residue, was found in the extract. Characteristics of the chemical composition of each batch were analyzed by thin layer and HPLC chromatograms to obtain the identity, and consistency of constituents fingerprint for controlling batch-to-batch variability. 

For *in vitro* treatment, ME was diluted in MEM, added to SK-N-SH cells at final concentrations ranging from 0 to 1,000 µg/ml, and incubated for 3 h prior to the addition of H_2_O_2_ or PCB-52 [17]. The cells were further incubated at 37°C for 24 h after which cytotoxicity was determined by MTT assay. Subsequently, optimal concentration of ME and preincubation time prior to the addition of H_2_O_2_ were chosen to obtain most effective protection by MTT assay.

### MTT assay

Cell viability was evaluated by spectrophotometric analysis using MTT [3-(4,5-dimethylthiazolyl-2-yl)-2,5-diphenyl-tetrazolium bromide] (Sigma-Aldrich; St. Louis, MO). SK-N-SH cells (2 X 10^5^ cells/ml) were grown overnight in 96-well plate with or without preincubation with ME for 3 h prior to H_2_O_2_ or PCB-52 (Accu Standard Inc., New Haven, CT) treatment. MTT solution was added to each well at a final concentration of 500 µg/ml. Formazan crystals formed by living cells were then dissolved in isopropanol and measured at 570 nm by Multi-Detection microplate reader (BioTek Instruments, Inc.; Winooski, VT). 

### Measurement of intracellular ROS levels

Intracellular ROS levels were quantified using 2′,7′-dichlorodihydrofluorescein diacetate (DCFH-DA) (Sigma-Aldrich; St. Louis, MO). The DCFH-DA probe was cleaved to H_2_DCF by cytoplasmic esterases and subsequently converted to a fluorescent DCF in the presence of intracellular ROS. SK-N-SH cells (2 X 10^5^ cells/ml) were seeded in 24-well plate and treated with H_2_O_2_ (150-600 µM) or PCB-52 (5-30 µg/ml) for 24 h with or without prior preincubation with 200-800 μg/ml ME. The cells were harvested, washed once with PBS followed by incubation with 50 µM DCFH-DA at 37°C in the dark for 45 min. The fluorescence intensity of DCF was quantified by Multi-Detection microplate reader at 485 nm excitation and 530 nm emission. ROS levels in mice brain extract (BE) were also measured using the same procedures except that 100 µl of diluted BE containing 5 mg protein was used instead of the cells.

### Determination of caspase-3 activity

Caspase-3 activity was determined using a colorimetric assay kit, CaspACE assay system (Promega Corporation; Madison, WI), according to the manufacturer’s instructions. The specific cleavage of substrate DEVD-pNA by caspase-3 was measured at 405 nm. The amounts of caspase-3 in the cells or brain extracts were expressed in relative to the amounts in the untreated control group.

### Assay for Acetylcholine Esterase (AChE) inhibition

SK-N-SH cells (10 x 10^5^ cells/ml) were plated in 12-well plate and incubated overnight at 37°C in 5% CO_2_. After an exposure to ME (200–600 µg/ml) or donepezil (200-600 μg/ml) (Pfizer Inc.; New York, NY) for 24 h, the cells were trypsinized and lysed by freezing and thawing. Cell lysate was centrifuged at 15,000 rpm at 4°C for 10 min and the supernatant was collected, assayed for protein by Pierce BCA protein assay kits (Thermo Scientific Inc.; Rockford, IL) and kept at -20°C until further used. AChE was measured in a 96-well microplate using the Amplex^®^ Red (10-acetyl-3, 7-dihydroxyphenoxazine) Acetylcholine/Acetylcholinesterase Assay Kit (Molecular Probes, Inc.; Eugene, OR). AChE converts acetylcholine substrate to choline, which is in turn oxidized by choline oxidase to betaine and H_2_O_2_. Finally, H_2_O_2_ reacts with Amplex Red reagent to generate the fluorescent resorufin, which can be measured by Multi-Detection microplate reader (BioTek Instruments) at 571 nm excitation and 585 nm emission wavelengths.

### Animal maintenance

Male ICR mice weighing between 25-35 g were obtained from the Animal’s House, Faculty of Medicine, Khon Kaen University, Khon Kaen, Thailand. The mice were housed in stainless steel cages in a group of 4-5, in an air-conditioned room maintained at 25±2 °C with a 12:12 h light: dark cycle, and fed with standard pellet diet (Charoen Pokphand Foods Public Company Ltd., Bangkok, Thailand) and water *ad libitum*. Eight to 10 mice at 8-10 weeks were used in each treatment group. All procedures were complied with the standards of care and use of experimental animals as approved by the Animal Ethics Committee, Faculty of Medicine, Khon Kaen University, Thailand, record number AE09/53.

### Spatial memory test

The test for spatial memory was modified from the Morris water maze test [18]. The open-field water maze consisted of a water-filled plastic pool (100 cm in diameter, 40 cm deep) in which a circular platform (10 cm in diameter) was submerged below the milky water surface at the center of one quadrant (the target quadrant). Water (control) or ME (50 or 100 mg/kg body weight), in a volume of 0.05 ml/10 g body weight, were given to mice once daily by gavage administration. On day 1, 2 and 3, the animals were subjected to the Morris water maze test 45 min after the treatment. The tests were performed 1 min/trial, 3 trials/day and with an inter-trial interval of 15 min. 

The effects of ME against scopolamine-induced amnesia in mice were also studied. Oral gavage was used to feed mice once daily with either water (control) or ME (100 mg/kg body weight) for 16 days. On day 14, 15 and 16, mice were injected subcutaneously with either water or scopolamine (1 mg/kg body weight) (Sigma-Aldrich, St. Louis, MO, USA) 15 min after ME/water feeding. Mice were subsequently subjected to the Morris water maze test 30 min after scopolamine injection.

### Fear memory: passive avoidance test

 The passive avoidance test was modified from the earlier reports [19-21]. In brief, the apparatus consisted of one clear and one dark chamber, separated by a guillotine door. The floor on the clear chamber (12×10×12 cm) and the dark chamber (12×10×12 cm) was composed of 2-mm stainless steel rods spaced at 0.5 cm apart. The apparatus was illuminated by fluorescent lamp. The step-through latency times were determined in mice underwent one training trial and three test trials 24 h apart. For the training trial, the animal was initially placed in the clear chamber. When the mouse entered the dark compartment, the door connecting both compartments was closed and an electrical foot shock (1.5 mA) of 2 sec duration (electroshock generator type 207, HSE HUGO SACHS, Germany) was delivered through the stainless steel rods. 

In this study, the mice were orally treated with either distilled water (as control) or ME (100 mg/kg body weight) once daily for 17 days. On day 14-17, the animals were subcutaneously injected with either distilled water or scopolamine (1 mg/kg body weight) 15 min after oral ME/water treatment. Thirty minutes after the injection, all mice were subjected to passive avoidance test as the training trial (day 14) and 3 test trials (day 15-17). The latency to enter the dark compartment was recorded up to 300 sec.

### Brain homogenate preparation

 At the end of passive avoidance test, mice were terminated by cervical dislocation and the whole brains were removed and kept at -20°C. Brain homogenate was prepared by removing the fibrous tissues and fat, then weighed, chopped, and homogenized in the Locke’s buffer (154 mM NaCl, 5.6 mM KCl, 3.6 mM NaHCO3, 2.0 mM CaCl2, 10 mM glucose, 5 mM HEPES, pH 7.4, 1 mM PMSF and 1 mM Leupeptin ) (Sigma-Aldrich; St. Louis, MO) [22]. The homogenates were measured for protein concentration using Pierce BCA protein assay kits (Thermo Scientific Inc.) after which each homogenate was diluted in Locke’s buffer to 50 mg/ml and kept at -20°C. A final concentration of 5 mg/ml of brain homogenate was used in each subsequent experiment. ROS levels and caspase-3 activities were analyzed within few days after the homogenization process. 

### Western blot analysis of KPNB1

A total of 30 µg proteins derived from the brain extracts were resolved by 12% SDS-PAGE and then transferred onto a nitrocellulose membrane using a semi-dry transfer apparatus (GE Healthcare; Uppsala, Sweden). Non-specific bindings were blocked with 5% skim milk in PBS for 1 h. The membrane was then incubated with mouse monoclonal anti-KPNB1 or anti-β-actin antibody (as the loading control) (both from Santa Cruz Biotechnology Inc.; CA, USA) with a dilution of 1:1,000 in 1% skim milk/PBS at 4°C overnight. After washing with PBS three times, the membrane was further incubated with rabbit anti-mouse IgG conjugated with horseradish peroxidase (1:2,000 in 1% skim milk/PBS) at room temperature for 1 h. Reactive protein bands were visualized with SuperSignal West Pico chemiluminescence substrate (Pierce Biotechnology Inc.; IL, USA) using autoradiogram. 

### Statistical analysis

All quantitative data were reported as mean ± standard error of mean (SEM). Statistical analyses were carried out using SPSS version 16.0 (SPSS Inc.; Chicago, IL). Multiple comparisons of more than two groups (n=3 for each group) of variables were performed by one-way analysis of variance (ANOVA) with Tukey post-hoc test, whereas comparisons between two groups of variables were performed by unpaired Student’s *t* test (when the data distributed normally) or nonparametric Mann-Whitney U test (when the data did not distribute normally). Statistical significance was considered when *P*-value ≤ 0.05.

## Results

### *In vitro* cytotoxicity test for ME and protective effects of ME against H_2_O_2_-induced cell death

**Figure 1:**
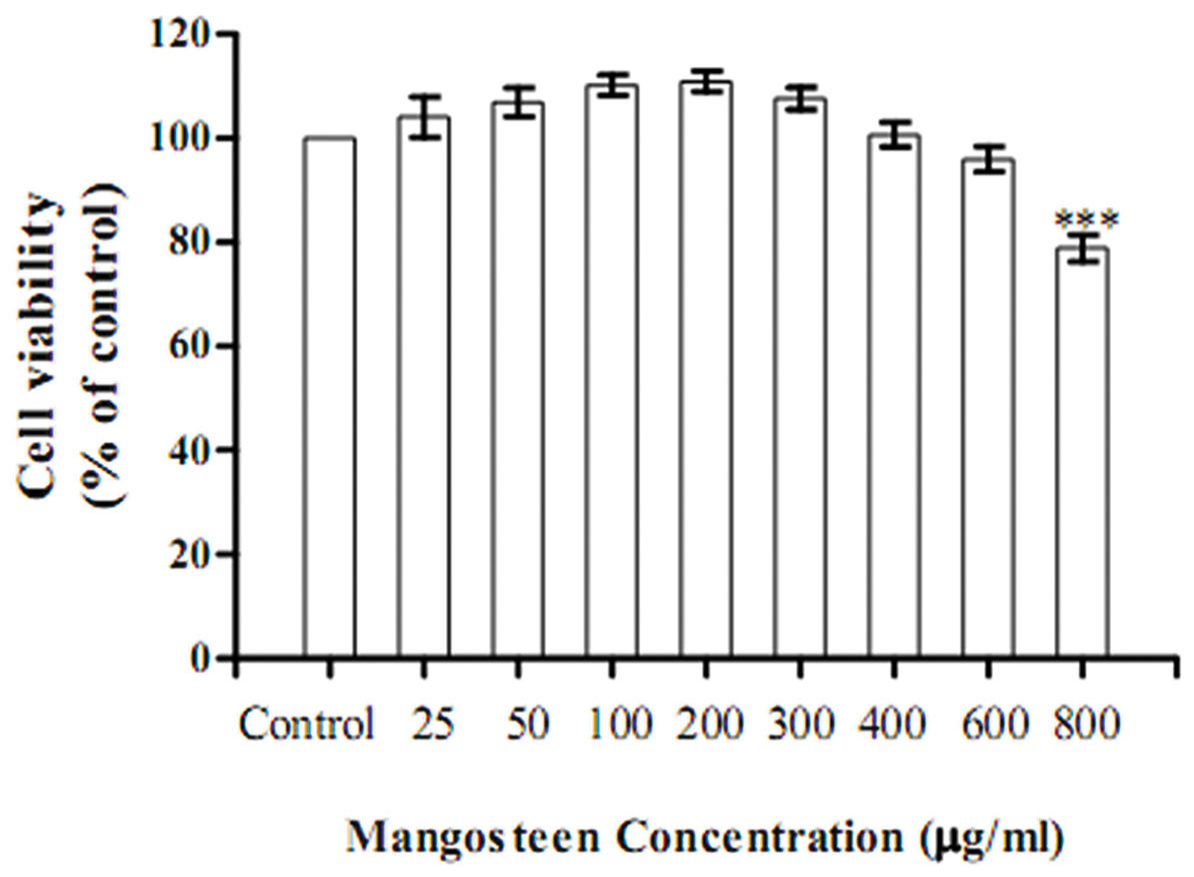
Cytotoxicity screening of mangosteen extract in SK-N-SH cells. SK-N-SH cells were treated with mangosteen extract at the concentrations of 25-800 µg/ml. After 24-hour incubation, cytotoxicity was determined by MTT assay. Data were presented as mean ± SEM (n=8 for each group). ***P < 0.001 compared to control.

Cytotoxicity screening of ME at 0-1,000 µg/ml concentrations on SK-N-SH neuroblastoma cells after 24-h incubation was determined by MTT assay. The amounts of viable cells were mildly decreased after the exposure of ME at 800 µg/ml (Figure **1**). Thus, the concentration of ME used in this study was limited up to 800 µg/ml. 

**Figure 2:**
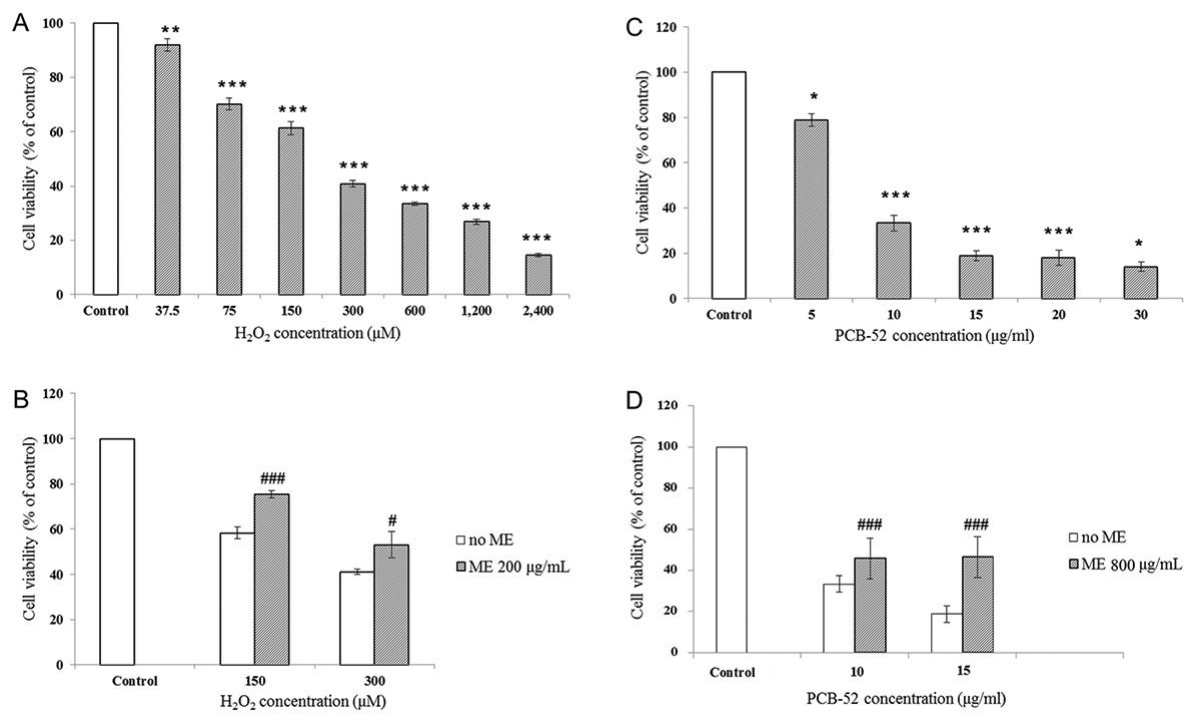
Effects of ME on chemical-induced cytotoxicity. (**A**) Cytotoxic effects of H_2_O_2_ on SK-N-SH cells after 24-h incubation. (**B**) Effects of ME on H_2_O_2_-induced cytotoxicity. (**C**) Cytotoxic effects of PCB-52 on SK-N-SH cells after 24-h incubation. (**D**) Effects of ME on PCB-52-induced cytotoxicity. SK-N-SH cells were preincubated with ME for 3 h before the treatment of H_2_O_2_ and PCB-52 for 24 h. Cell viability was determined by MTT assay and expressed as percentage compared to untreated cells. Data were reported as mean ± SEM (n=10 independent experiments for H_2_O_2_). *P < 0.05, ** *P* < 0.01, *** *P* < 0.001 compared to untreated control; ^#^ *P* < 0.05, ^###^ *P* < 0.001 compared to cells treated with corresponding chemicals concentration with no ME preincubation.

 H_2_O_2_ demonstrated dose-dependent cytotoxic effects on SK-N-SH cells after 24-h incubation (Figure **2A**). Exposure cells with H_2_O_2_ at 37.5 µM concentration reduced cell viability to 85.6% as compared to untreated cells. The cell viability progressively decreased to 7.9% in the cells treated with 2,400 µM H_2_O_2_. H_2_O_2_ at 150 µM appeared to be the optimal concentration to study protective effects of ME (roughly 57% viability). This concentration of H_2_O_2_ was therefore used in all subsequent experiments. Likewise, PCB-52 exposure also resulted in dose-dependent reduction in cell viability from 78.88% (compared to untreated cells) in 5 µg/ml exposure group to 14.04%. in 30 μg/ml exposure. The optimal PCB-52 concentration (10 μg/ml) was subsequently used in all subsequent experiments.

Preincubation of SK-N-SH cells with 25-800 μg/ml ME for 3 h successfully prevented the cytotoxic effect of H_2_O_2_. The highest protective effect of ME at 200 µg/ml significantly ameliorated cytotoxic effect of 150 μM H_2_O_2_ (Figure **2A and 2B**) while 800 μg/ml of ME provided the best protection against 10 μg/ml PCB-52 (Figure **2C and 2D**). Therefore, the two concentrations of ME were used in all subsequent experiments. 

### *In vitro* effects of ME on oxidative stress induced by H_2_O_2_ and PCB-52

**Figure 3:**
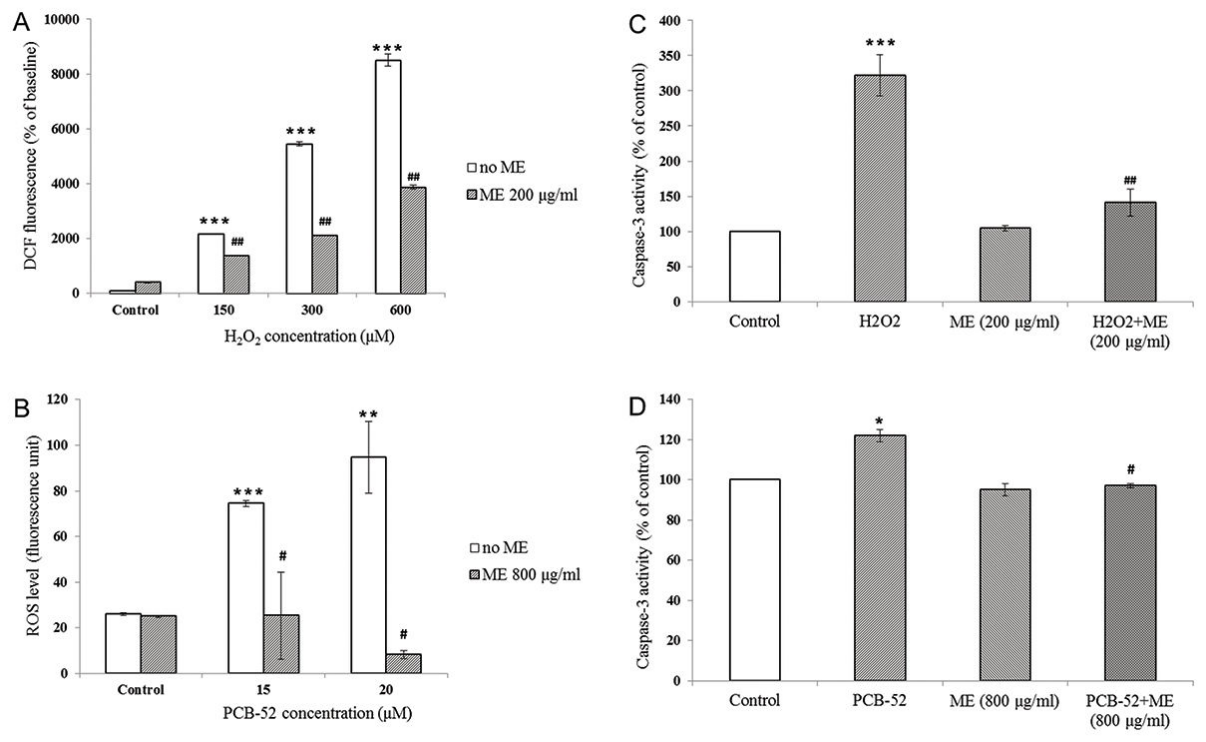
Effects of ME on oxidative stress and apoptosis *in* *vitro*. (**A**, **B**) Intracellular levels of ROS in SK-N-SH cells treated with H_2_O_2_ (**A**) or PCB-52 (**B**) for 24 h with or without prior ME incubation. ROS levels were determined using DCFH-DA assay. The fluorescence was measured and expressed as percentage compared to untreated cells (n=3 independent experiments for each bar). (**C**, **D**) Caspase-3 activity in SK-N-SH cells treated with H_2_O_2_ (**C**) or PCB-52 (**D**) with or without ME preincubation. Caspase-3 activity was measured using colorimetric assay. Data were expressed as percentage of the activity compared to untreated cells (n=5 independent experiments for each bar). *P < 0.05, ** *P* < 0.01, *** *P* < 0.001 compared to untreated control; ^#^ *P* < 0.05, ^##^ *P* < 0.01 compared to cells treated with corresponding chemicals concentration with no ME preincubation.

The cellular oxidative stress was evaluated through the measurement of intracellular ROS levels. As shown in Figure **3A**, the exposure of SK-N-SH cells to H_2_O_2_ at 150, 300 and 600 µM concentrations significantly increased intracellular ROS levels to 21, 54 and 86 folds compared to untreated cells, respectively. Preincubation of SK-N-SH cells with 200 μg/ml of ME significantly decreased H_2_O_2_-induced intracellular ROS levels regardless of H_2_O_2_ concentrations (Figure **3C**). Similarly, treatment of PCB-52 (Figure **3B**) at 15-20 μg/ml concentrations increased intracellular ROS levels by 3.5-4.5 fold which were normalized by preincubation of 800 μg/ml ME (Figure **3D**). Fluorescence images of SK-N-SH cells stained with Hoechst 33342 after 24-h exposure to H_2_O_2_, PCB-52 and Aβ, with and without ME preincubation were taken under inverted confocal microscopy. ME at 200, 800 and 400 μg/ml demonstrated the highest protective effect against H_2_O_2_, PCB-52 and Aβ toxicity, respectively (Figure **S1**).

### *In vitro* effects of ME on caspase-3 activity

Caspase-3 activity was expressed as fold-change compared to untreated controls. Preincubation of 200 μg/ml ME significantly blunted inductive effects of 150 µM H_2_O_2_ on caspase-3 activity. As shown in Figure **3C**, ME preincubation significantly suppressed the effects of 150 µM H_2_O_2_ on caspase-3 activity by 56% (from 3.2 folds to 1.4 folds). Similar findings were also found in cell treated with PCB-52 (Figure **3D**). 

### *In vitro* effects of ME on Acetylcholinesterase (AChE) inhibition

**Figure 4:**
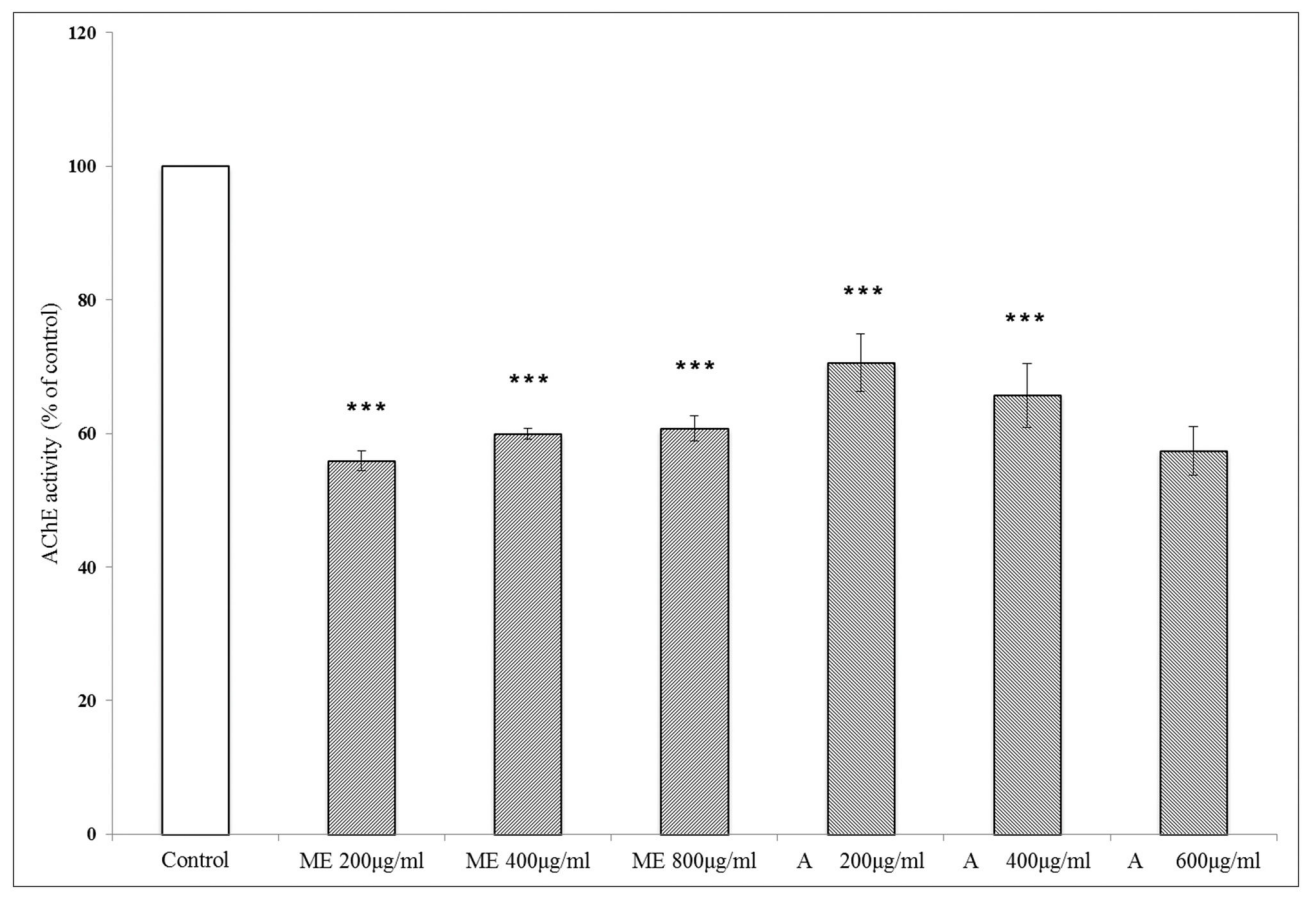
Effects of ME on AChE activity. AChE activity was determined in SK-N-SH cells treated with ME or Donepezil (Aricept^®^) (**A**) at different concentration as stated on the X-axis for 24 h. The activity was measured using Amplex^®^ Red Acetylcholine/Acetylcholinesterase Assay Kit and expressed as percentage compared to untreated cells. Data were reported as Mean ± SEM (n=3 independent experiments for each bar). *** *P* < 0.001 compared to untreated control.

 AChE activity was measured by acetylcholine/acetylcholinesterase assay kit. Treatment of ME at concentration either of 200, 400 or 800 µg/ml in SK-N-SH cells significantly inhibited AChE activity to roughly 60% compared to controls (Figure **4**). Treatment of the cells with donepezil, a drug for early AD patients, at the levels of 200, 400 or 600 μg/ml also yielded similar inhibitory effects on AChE activity.

### Effect of ME on spatial memory: Morris water maze test

**Figure 5:**
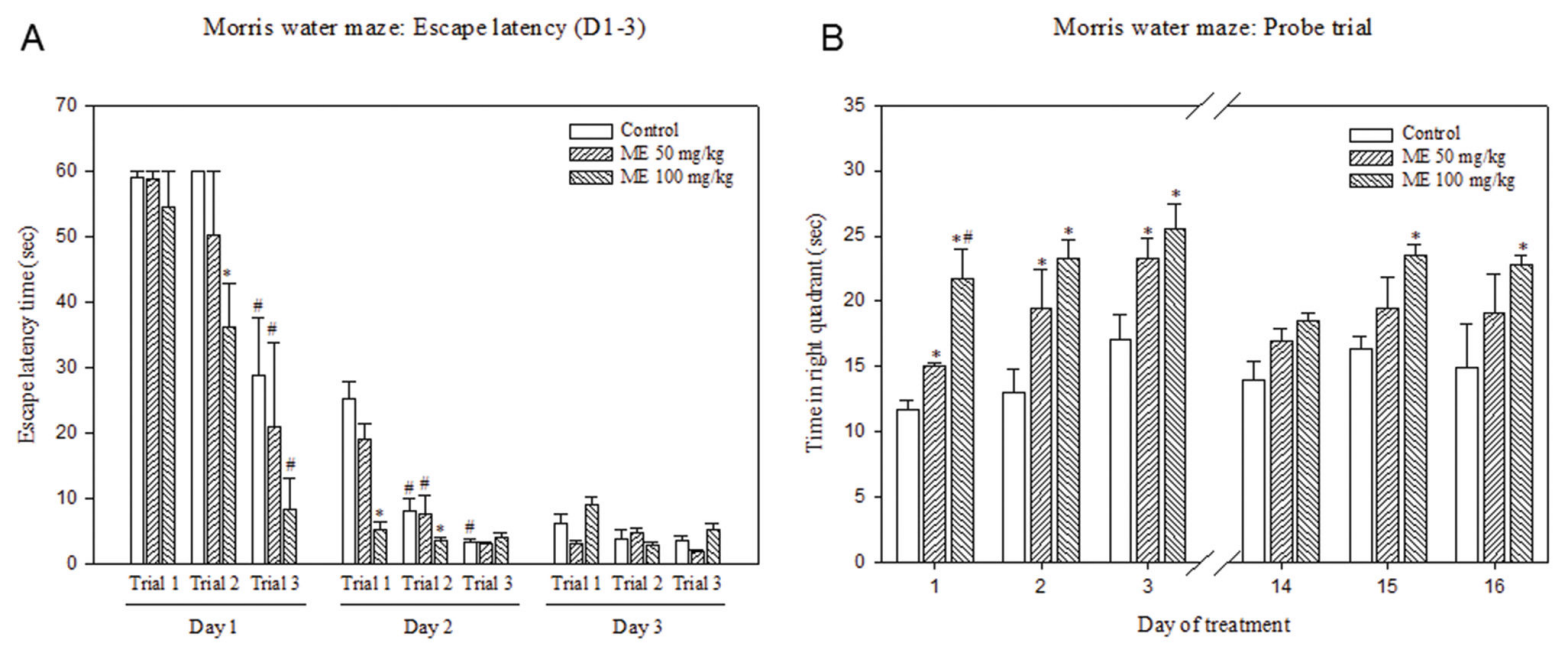
Effects of ME on spatial memory in mice. (**A**) Effects of ME on escape latency during 3 test trials of day1, 2 and 3 of the treatment using Morris water maze test. ME (50 or 100 mg/kg) or water (control) was orally administered to the mice 45 min before the trials (n=6). The escape latency (sec) was expressed as Mean ± SEM. * *P* < 0.05 compared to control in the same test trial on the same test day; # *P* <0.05 compared to the trial 1 of the same test condition in the same test day. (**B**) Effects of ME on time in right quadrant (probe trial) on day 1-3 and 14-16 of the treatment using Morris water maze test. The mice were orally administered with water (control) or ME (50 and 100 mg/kg) at 45 minutes before the test. The escape latency (sec) was expressed as Mean ± SEM (n=6). * *P* < 0.05 compared to control on the same test day; # *P* < 0.05 compared to 50 mg/kg ME-treated group on the same test day.

 Control mice showed the ability to learn as seen by a significantly decreased in escape latency of trial 3 compared to trial 1 on day 1 as well as significantly reduced escape latency of trials 2 and 3 compared to trial 1 on day 2 (Figure **5A**). Mice received 50 mg/kg ME showed no difference in escape latency compared to the control. Mice treated with ME 100 mg/kg demonstrated increased learning ability as suggested by significant reductions in escape latency of trials 1 and 2 on day 1 and 2 when compared to the control in the same trial of the same day. However, on day 3, no difference in escape latency among the groups could be observed. On day 14-16 of the treatment, the escape latency times in each group were in similar pattern as those observed during day 1-3 (Figure **5B**). Likewise, the significant effect of 100 mg/kg ME in reducing escape latency was observed. 

**Figure 6:**
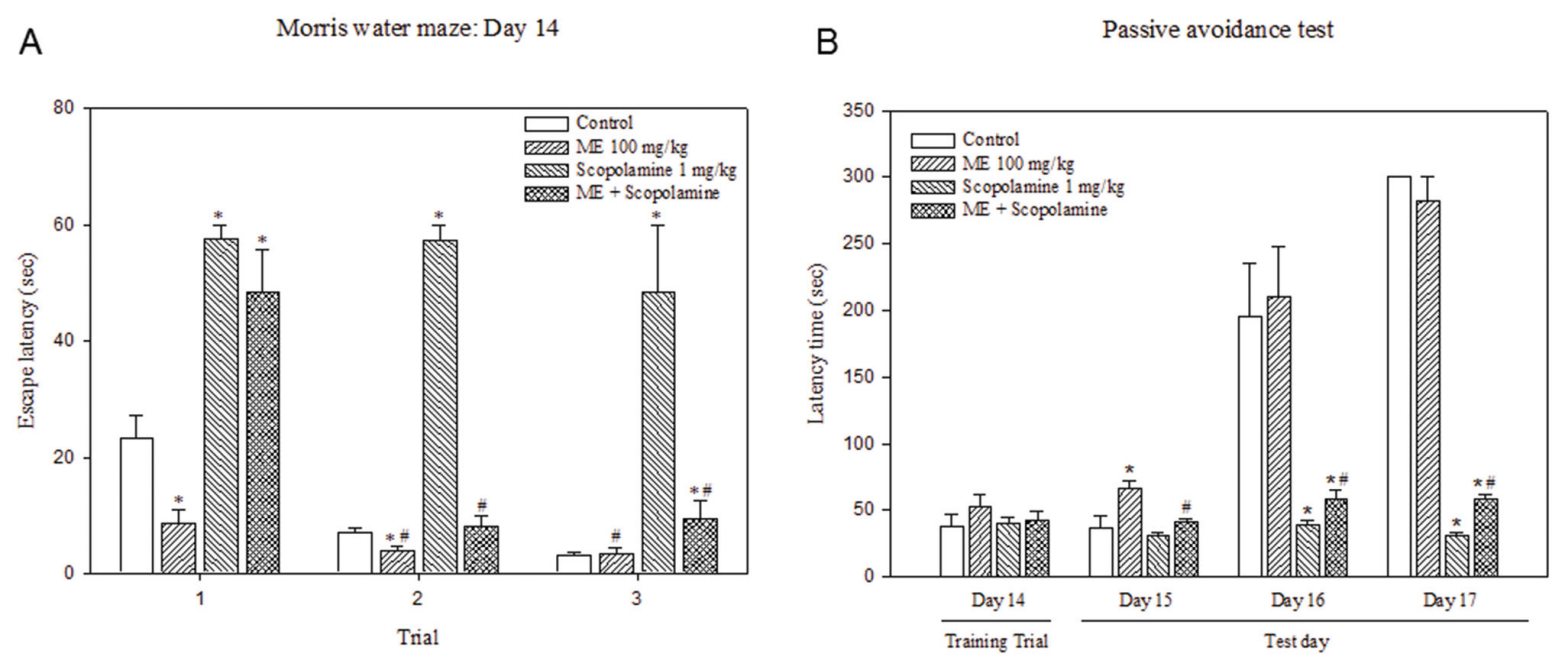
Effects of ME on scopolamine-induced memory impairment in mice. Mice were orally administered with water (control) or 100 mg/kg ME once daily for 14 days. On day 14, the mice were subcutaneously injected with water or 1 mg/kg scopolamine 15 min after water/ME administration. The mice were subsequently subjected to Morris water maze test (**A**) or passive avoidance test (**B**) at 30 min after the injection. Latency time (sec) was expressed as Mean ± SEM (sec) (n=6). * *P* < 0.05 compared to control on the same test day; # *P* < 0.05 compared to scopolamine-treated group on the same test day.

Scopolamine-induced memory deficit was also attenuated by pretreatment of 100 mg/kg ME for 14 days (Figure **6A**). Scopolamine treatment in mice resulted in a significant increase in escape latency times in all trials indicating memory impairment. Pretreatment of ME for 14 days prevented the impairment effects of scopolamine on memory as evidenced by a significant decrease in escape latency time (trials 2 and 3) in ME-pretreated group compared to scopolamine-treated group with no prior ME treatment.

### Effect of ME on fear memory: Passive avoidance test

Effects of ME on scopolamine-induced impaired passive avoidance test were determined. As shown in Figure **6B**, no difference in step-through latencies was detected among all groups in training trial (day 14). On the first day of the test (day 15), mice treated with 100 mg/kg ME had a significantly longer step-through latency time than control suggesting retained of fear memory. On test day 2 and 3 (day 16 and 17), the step-through latency times were significantly shorter in scopolamine-treated mice than the control mice. Effects of scopolamine on latency time were partially abrogated by the pretreatment of ME as suggested by significantly longer latency times in mice pretreated with ME compared to non-pretreated mice. 

### Effect of ME on ROS levels and caspase-3 activity in brain extract (BE) from scopolamine-treated mice

**Figure 7:**
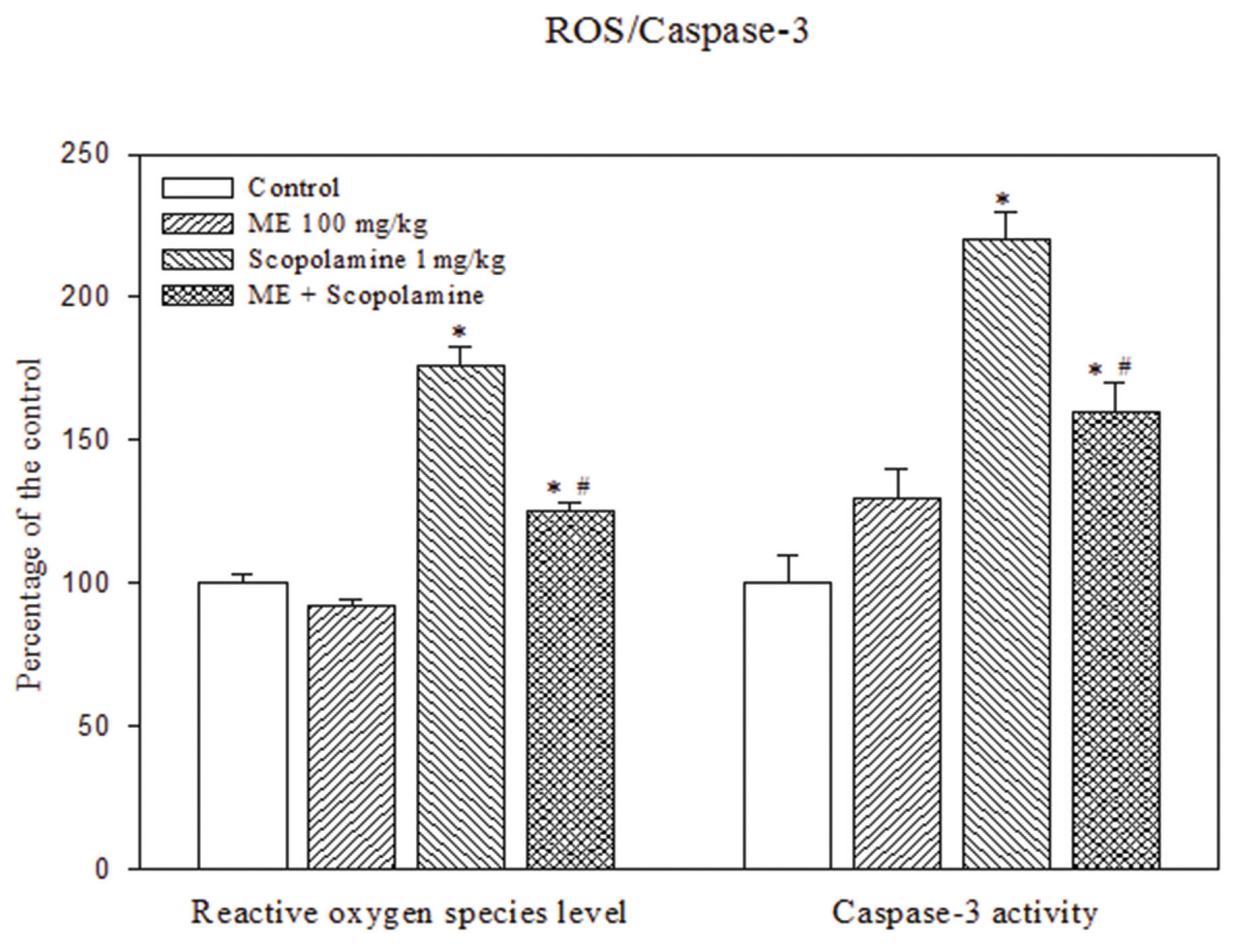
Effects of ME on reactive oxygen species (ROS) level and caspase-3 activity in mouse brain homogenates. ROS levels and caspase-3 activity were determined in brain homogenates of water/scopolamine-treated mice with/without prior ME treatment for 14 days. Data were presented as percentage compared to untreated cells and reported as Mean ± SEM (n=6). * *P* < 0.05 compared to the control; ^#^ *P* < 0.05 compared to scopolamine- treated group.

 BE from scopolamine-treated mice contained significantly higher ROS levels and caspase-3 activity compared to the control mice. Pretreatment of 100 mg/kg ME for 14 days significantly reduced ROS levels by roughly 30% (from 17.6 to 12.5 folds relative to control mice) (Figure **7**). Similarly, ME pretreatment also resulted in amelioration of scopolamine-mediated caspase-3 induction (from 2.2 to 1.6 folds relative to untreated mice). 

### Effect of ME on Karyopherin β1 (KPNB1) level in the brain of scopolamine-treated mice

**Figure 8:**
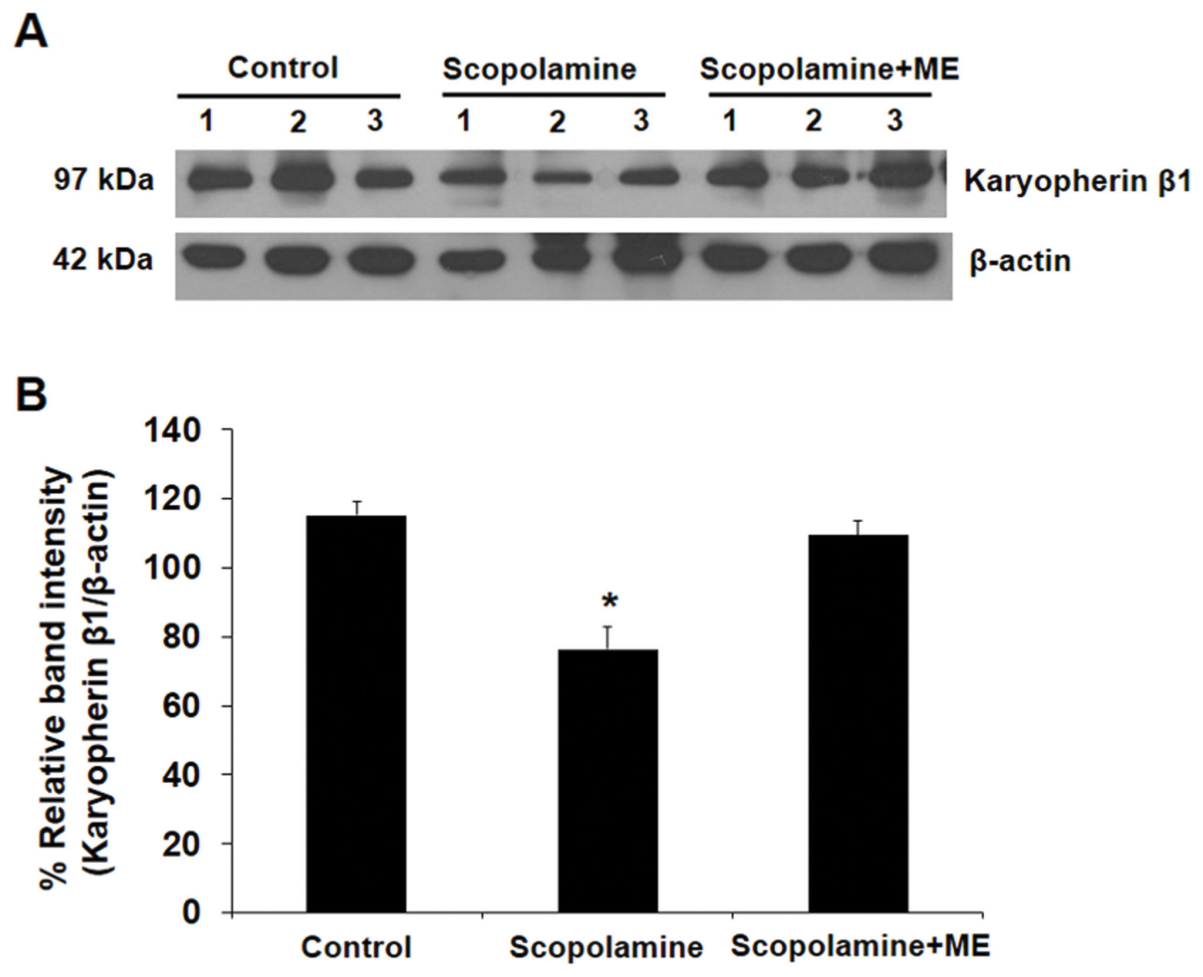
Western blot analysis of karyopherin β1 (KPNB1) in mouse brain homogenates. Scopolamine-treated mice with or without prior ME treatment were obtained from passive avoidance test. At the end of the test, whole brain from the mice was subjected to protein extraction. A total 30 µg proteins derived from brain extracts (n=3) were resolved by 12% SDS-PAGE and transferred onto a nitrocellulose membrane. After blocking non-specific bindings, the membrane was incubated with mouse monoclonal anti-KPNB1 (1:1,000 in 1% skim milk/PBS) and then incubated with rabbit anti-mouse IgG conjugated with horseradish peroxidase (1:2,000 in 1% skim milk/PBS). β-actin served as the loading control. The immunoreactive bands were visualized by chemiluminescence and autoradiography. Data was presented as KPNB1 band intensity relative to β-actin band. KPNB1 (97 kDa) level was significantly decreased after treatment with scopolamine but could be successfully preserved at its basal level by mangosteen extract. Data were reported as Mean ± SEM (n=3). * *P* < 0.05 compared to the control.

We further explored the effects of ME on the expression of karyopherin β1 (KPNB1), a protein involving in importing key molecules through nuclear compartment. KPNB1 is a cargo protein that binds to cytoplasmic proteins containing nuclear localizing signals (NLS). Together with karyopherin α2 (KPNA2), cytoplasmic proteins bind to KPNB1 and KPNA2 heterodimer, attach to nuclear pore complex, then get translocate inside the nucleus. Hence, KPNB1 can be found both in the nucleus, on nuclear membrane, and cytoplasm [23]. According to our previous study, KPNB1 expression was markedly decreased in SK-N-SH human neuroblastoma cell line after Aβ treatment and such reduced KPNB1 expression could be prevented by ME [14]. In the present study, we thus confirmed the previously reported findings *in vivo* through Western blot analysis of KPNB1 in brain extracts from scopolamine-treated mice with or without ME pretreatment. As shown in Figure **8**, we found that KPNB1 level was markedly reduced in scopolamine-treated mice and ME pretreatment successfully preserved KPNB1 at its basal level.

### Toxicity study of repeated ME administration

 Chronic toxicity of ME were determined in mice orally treated with either 50 or 500 mg/kg ME once daily for 14, 28, 56 or 84 days. The mice showed no sign of abnormality during the entire course of the treatment. In addition, both gross and microscopic examination revealed no pathological change in the stomach, heart, liver, kidney, spleen and testes (data not shown).

## Discussion

Oxidative stress has been implicated in the progression of a number of neurodegenerative diseases, including dementia [24]. High correlations between oxidative stress and cognitive impairment have been reported suggesting that oxidative stress should be considered as one of the risk factors in the development of cognitive disorders [24]. Since oxidative stress seems to be involved in the earliest phases of AD, the administration of antioxidants may be useful in the prevention and treatment of AD and other neurodegenerative disorders [25]. 

Many studies have reported that ME possesses antioxidant properties [26-28]. Xanthones isolated from mangosteen showed antioxidative properties, especially the xanthones that were isolated from pericarp. Among the isolated xanthones, 8 hydroxycudraxanthone G, gartanin, α-mangostin, γ-mangostin and smeathxanthone A, were compounds with the highest antioxidant activities [29]. Marquez-Valadez et al. showed that α-mangostin exerted a robust anti-peroxidative effect in brain tissue preparations probably through its properties as a free radical scavenger [30]. In addition, Pedraza-Chaverri et al. suggested that α-mangostin was able to directly scavenge ROS and has neuroprotective effects against 3-nitropropionic acid in primary cultures of cerebellar granule neurons [15]. The same study also demonstrated an ability of α-mangostin to ameliorate 3-nitropropionic acid-induced ROS production. In summary, the aforementioned studies showed the importance of xanthones in reducing oxidative damage in the brain. 

α-Mangostin is the major constituent in mangosteen pericarp, especially from crude ethanolic extract. However, our previous study found that the amount of α-mangostin in ME (water extract part) must be less than 2%, since α-mangostin was toxic to cells despite its high antioxidative activity. In agreement, we observed that crude ethanol ME, containing approximately 10% α-mangostin, possessed high cytotoxic effects in many cell lines and demonstrated antitumor progression in tumor-bearing mice [16,30]. Moreover, the purified α-mangostin (isolated from mangosteen or from commercially available compound; Chengdu Biopurify Phytochemicals Ltd., Sichuan, China) demonstrated both cytotoxic and apoptotic effects in several cancer cell lines as measured by MTT assay and flow cytometry (data not shown). Therefore, we determined cytotoxicity and ROS scavenging property of the water-extracted ME utilized in the present study in order to obtain the most effective concentration for neuroprotective effects. The preparation of partially purified water ME contained <2% α-mangostin with several polyphenolic compounds that possessed high antioxidative activity and very low cytotoxic activity in MTT assay at IC_50_ > 150 µg/ml.

Caspase activation is a major and early event in apoptotic process. Among all members of caspases, caspase-3 is the major executor of apoptosis and its activation has been shown to be a critical event in neuronal apoptosis. Notably, the levels of caspase-3 and caspase-3-degraded product were increased in AD brains [31-33]. In our previous study, the protective effects of ME against Aβ-induced cytotoxicity were confirmed in SK-N-SH cells as suggested by the suppression of ROS levels and caspase-3 activity. The present study therefore, aimed to further delineate the protection of ME against H_2_O_2_ cytotoxicity. We firstly demonstrated that H_2_O_2_-induced cytotoxicity in SK-N-SH cells in a dose-dependent manner. The results were consistent with previous studies which showed that both chemicals caused apoptosis in cultured cells through the elevation of ROS and caspase-3 levels [15,34]. Interestingly, the H_2_O_2_-mediated increased ROS and caspase-3 levels were significantly prevented by preincubating the cells with 50-300 μg/ml ME prior to H_2_O_2_ induction. These data, therefore, strongly supported the protective effects of ME against H_2_O_2_ cytotoxicity.

In addition to H_2_O_2_, protective effects of ME was also studied in cell treated with PCBs which are harmful environmental chemicals widely used in various industries. The chemicals remain mainly in soils and water but occasionally in air. Chronic exposure to PCB-52 can induce cellular oxidative stress and apoptosis especially in neurons resulting in progressive memory cell loss and finally dementia [35]. In the present study, we also demonstrated that ME provided protection against PCB-52 in SK-N-SH cells. These findings demonstrated protective effects of ME in both endogenous cellular oxidative stress model (H_2_O_2_ treatment) and environmental toxic pollutant model (PCB-52).

According to the protective effects of ME against oxidative damage, mouse studies were then pursued in order to explore its *in vivo* benefits. We found that the treatment of ME at the doses of 50 or 500 mg/kg for up to 84 days had no observable toxicity in mice. These were in accordance with previous reports which found no toxicity in mice treated with hydroethanolic extract from mangosteen pericarp [26,36-38]. Intragastric administration of this extract at a single dose of 2 or 5 g/kg body weight produced no toxic signs during 14 days of observation. In addition, Jujun et al. [39]. showed that oral administration of 2, 3 or 5 g/kg body weight of crude mangosteen extract showed no toxicity, mortality, or adverse effect on growth rate [39]. The chronic toxicity of *Garcinia mangostana* L. pericarp extract in rat has also been previously reported. The study confirmed that, rats receiving mangosteen at 500 mg/kg/day for 6 months showed unaffected behavior, health status, hematological parameters nor any clinical manifestations. Also histopathological results of visceral organs revealed no significant lesion in spite of high content of 24.4% of α-mangostin in mangosteen [33] compared to a low level of 2% in water extract ME from our study. Since our experimental animals gave similar results of clinical and gross specimens findings therefore, we did not process on immunocytochemical study of the organs. 

Acetylcholine has been proposed to be one of the important neurotransmitters involved in learning and memory processes [6]. Our study in SK-N-SH cells showed that ME at concentrations between 200-800 µg/ml significantly suppressed the activity of AChE, which degrades acethycholine, to roughly 60% compared to controls. The results were comparable to effects of donepezil or Aricept at therapeutic levels (Figure **4**). Donepezil inhibits acetylcholinesterase, leading to an increase in acetylcholine levels in the brain and improved memory impairment in AD patients [7]. We, therefore, pursued *in vivo* studies utilizing scopolamine-induced amnesia mouse model. Scopolamine which is a muscarinic antagonist, has been shown to have many effects on brain, including increases AChE and malondialdehyde (MDA) levels in the cortex and hippocampus and abolish cerebral blood flow which was recovered by administration of (AChEIs) [40,41]. 

Our *in vivo* findings demonstrated that the aqueous extract of ME could enhance learning and memory in mice. The ME improved learning and memory abilities as well as attenuated scopolamine-induced amnesia in mice. The results were clearly evidenced in both models of spatial (Morris water maze test) and fear (passive avoidance test) memory. These results suggested that ME could improve memory impairment caused by central cholinergic nervous system dysfunction and might be mediated, in part, through cholinergic system. Hence, the effects of ME on cholinergic system and acetylcholine signaling pathway warrants further investigation. Furthermore, ME treatment was also found to ameliorate scopolamine-induced increases in caspase-3 activity and ROS levels in the brain of the mice. 

 In addition to acetylcholine, several proteins might be involved in the pathogenesis of neurodegeneration including KPNB1 which involves in nuclear import of macromolecule and neuronal differentiation processes [37]. The lack of KPNB1 has been reported in Aβ-treated cells as well as varieties of mental and neurodegenerative diseases [14,37]. Our previous proteomic analysis in SK-N-SH cells found that the levels of KPNB1 protein was markedly reduced in SK-N-SH cells treated with Aβ (1-42) and the KPNB1 expression was completely preserved by ME pre-incubation [16]. Consistently, our present *in vivo* study also demonstrated protective effect of ME on KPNB1 protein against scopolamine treatment in mice. These data support the role of ME in the protection of KPNB1 reduction. The Western blot results demonstrated that the total level of KPNB1 from whole brain tissue extracts remained unchanged when ME was administered to mice. However, we could not rule out the possibility that the nuclear/cytoplasmic ratio of KPNB1 might have change as a result of the cytotoxicity induced by ROS or PCB-52. These present data may lead to further identification of new therapeutic targets for treatment and prevention of AD. However, the exact mechanism(s) of such protective effects of ME remains to be elucidated. 

In conclusion, our study demonstrated protective effects of ME against toxic chemicals both *in vitro* and *in vivo* through antioxidant and AChE inhibitory properties. Moreover, our data suggests that ME could potentially be used as a cognitive enhancer, although further studies are required to delineate and explore the active principles in ME along with their mechanism(s) of action as well as their clinical safety and subsequent clinical trials.

## Supporting Information

Figure S1 **Fluorescence images of SK-N-SH cells stained with Hoechst 33342 after 24 h of exposure to H_2_O_2_, PCB-52 and Beta-amyloid, with and without ME preincubation.** ME at 200, 800 and 400 μg/ml demonstrated the highest protective effect against H_2_O_2_, PCB-52 and Beta-amyloid (Aβ) toxicity. Cells were observed under confocal fluorescence microscope.
(TIF)
