# Supplementary figures and images for: Protective Effects of Mangosteen Extract on H2O2-Induced Cytotoxicity in SK-N-SH Cells and Scopolamine-Induced Memory Impairment in Mice
Source: PLoS One. 2013 Dec 27;8(12):e85053. doi: 10.1371/journal.pone.0085053 (PMC3874002; doi:10.1371/journal.pone.0085053)

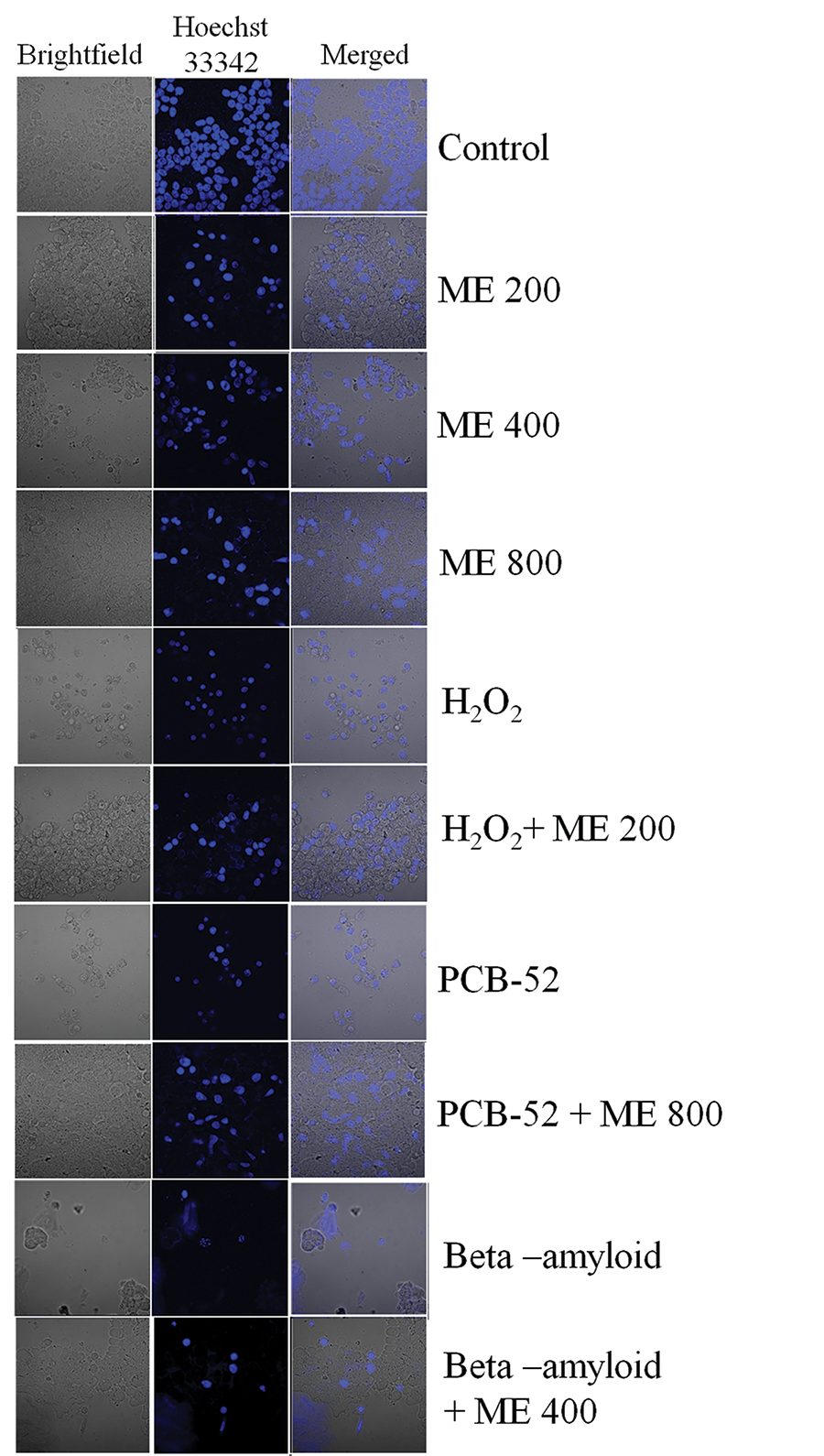

Supplement: Figure S1 — Fluorescence images of SK-N-SH cells stained with Hoechst 33342 after 24 h of exposure to H2O2, PCB-52 and Beta-amyloid, with and without ME preincubation. ME at 200, 800 and 400 μg/ml demonstrated the highest protective effect against H2O2, PCB-52 and Beta-amyloid (Aβ) toxicity. Cells were observed under confocal fluorescence microscope. (TIF) [file pone.0085053.s001.tif]
